# Supplementary material for: FERARI and cargo adaptors coordinate cargo flow through sorting endosomes
Source: Nat Commun. 2022 Aug 8;13:4620. doi: 10.1038/s41467-022-32377-y (PMC9359993; doi:10.1038/s41467-022-32377-y)
Supplement: Supplementary file 1 — Supplementary Information [file 41467_2022_32377_MOESM1_ESM.pdf]

Supplementary Figure 1

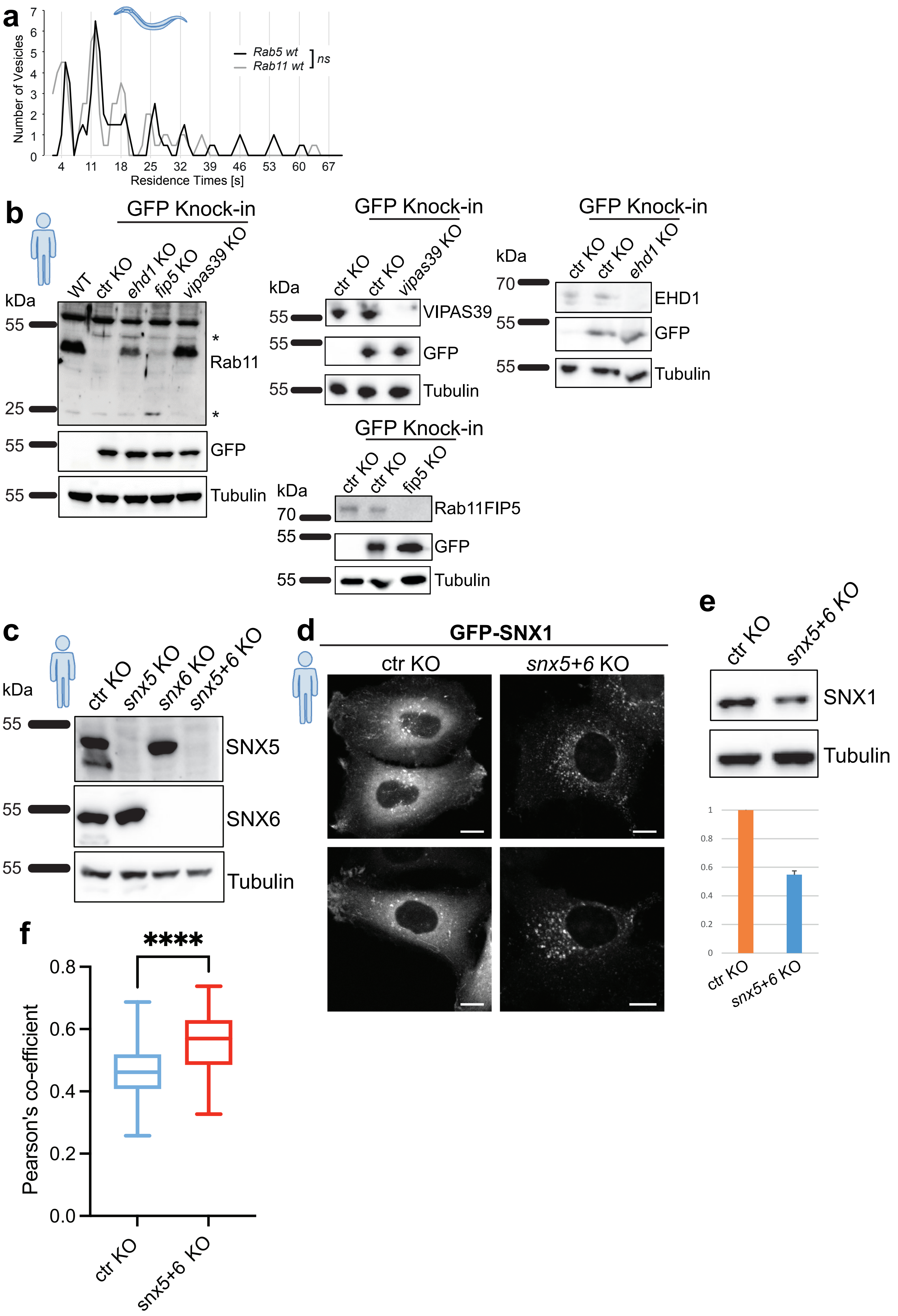

Supplementary Figure 1: (a) Comparison of vesicle residence times of Rab5 vesicles from this study with previously published Rab11 vesicles [7]. Unpaired two-tailed t test P=0.0652. (b) Western blots showing the GFP-Rab11 knock-ins of HeLa cells as indicated. Asterisks indicate unspecific bands. Second and third panel confirming FERARI knockouts (*vipas39, ehd1* and *rab11fip5*) in endogenously tagged GFP-Rab11 cell lines. (c) Western blot data showing the CRISPR/Cas9-mediated KO of *snx5*, *snx6* and combined KO of *snx5+6* detected by indicated antibodies. Tubulin was also detected to show equal loading. (d) Live images of ctr KO and *snx5+6* KO transiently over-expressing GFP-SNX1 (n=3 experiments with n=20 images each). Tubular structure of SNX1 is less in *snx5+6* KO cells in comparison with ctr KO cells. Unpaired two-tailed t test P=0.0131. Scale bars: 10 µm. (e) Western blot of HeLa cell extracts showing around 40% reduction of SNX1 protein level in *snx5+6* double knock-out cells. (f) Pearson's coefficient showing co-localization between GFP-SNX1 and mApple-Rab5. Data are presented as median with 25 to 75 percentile box and min to max whiskers. Rab5 co-localization with SNX1 is increased in *snx5+6* KO cells in comparison with ctr KO cells. Imaging data of this graph is shown in Fig. 6g. Unpaired two-tailed t test P=1.064E-05.

Supplementary Figure 2

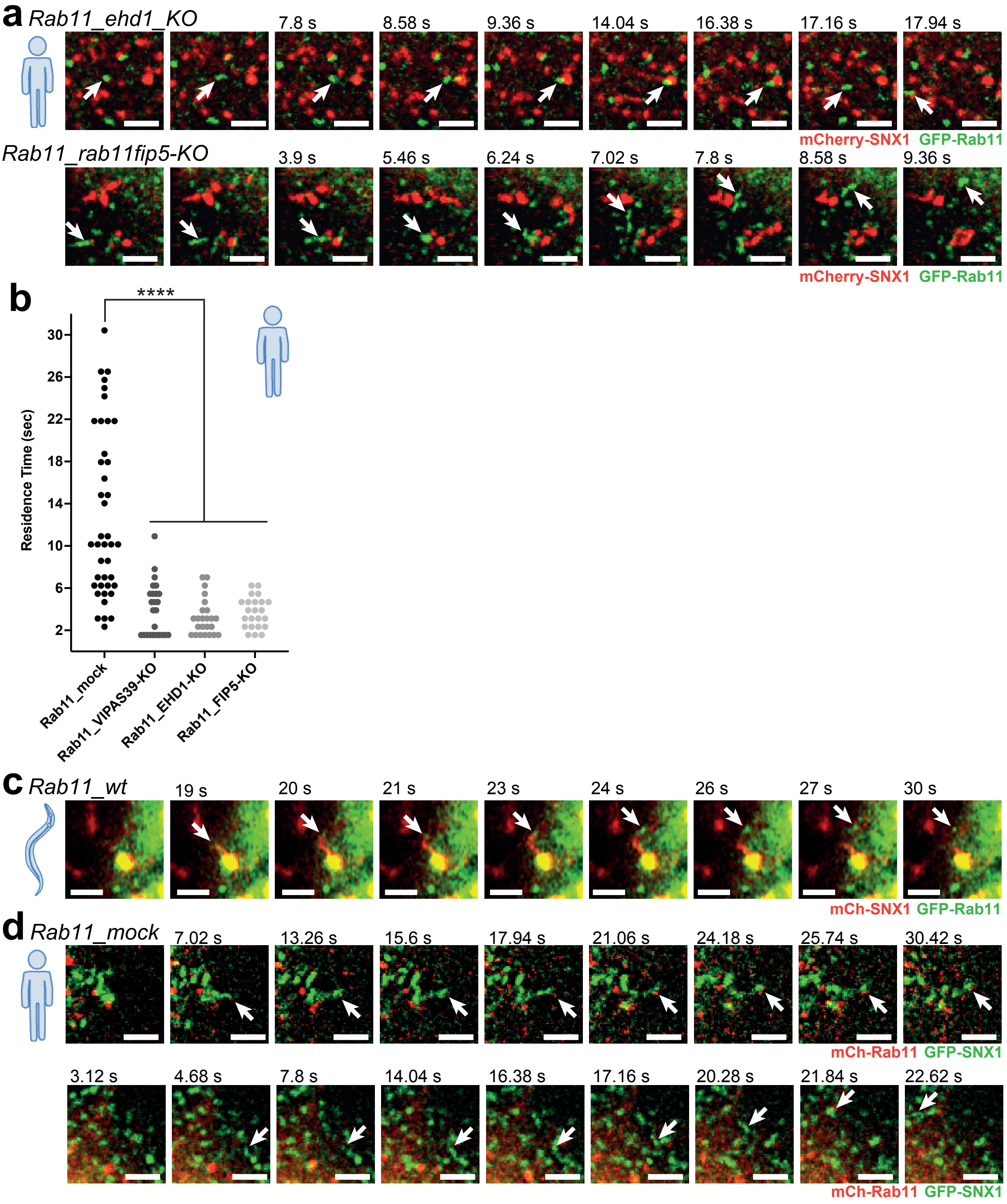

Supplementary Figure 2: (a) Movie stills showing kiss-and-run of endogenously tagged GFP-Rab11 vesicles (arrow) in *ehd1-KO* (n=24) and *rab11fip5*-KO (n=23) HeLa cells (see also Supplementary Movie 1). Scale bar: 2 µm. (b) Rab11 vesicles dock on SNX1 networks with residence times in distinct intervals. This pattern is abolished in *vipas39, ehd-1* and *rab11fip5* knock-out cells (see Fig. 1a, b). P values are given in Fig. 1b. (c) Movie stills (from Supplementary Movie 2) showing tubule formation from a sorting endosome and a Rab11 vesicle pinching off from the tubule (arrow) in *C. elegans* (n=9 vesicles). Scale bar: 2 µm. (d) Two series of movie stills (from Supplementary Movie 2) showing the same vesicle biogenesis process as in (c) in HeLa cells (n=10 vesicles). Scale bar: 2 µm.

Supplementary Figure 3

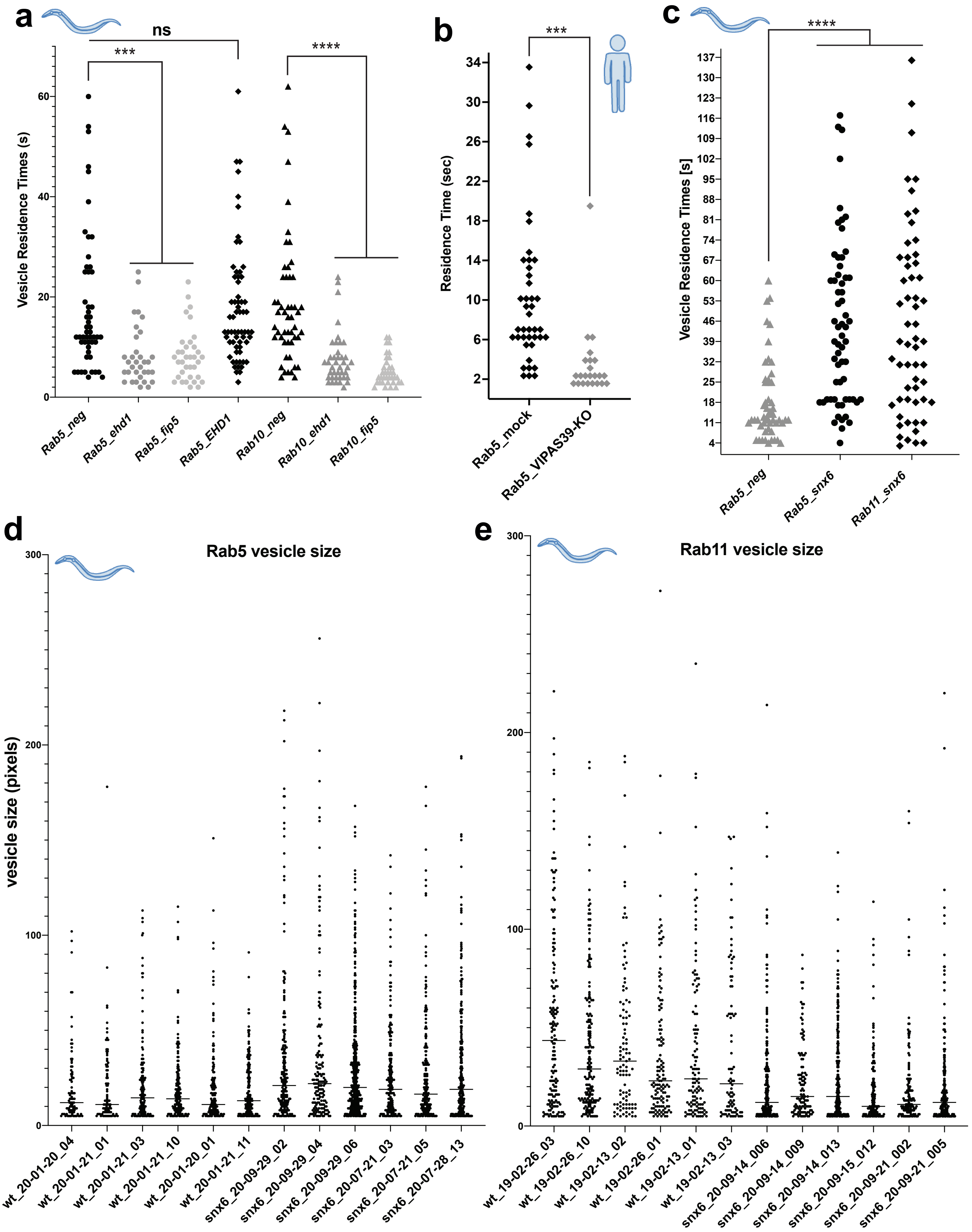

Supplementary Figure 3: (a) Single vesicle plots for kiss-and-run vesicles. These graphs are a different representation of the data in Fig. 1f, g (Rab5) and Fig. 3f (Rab10) containing all the single vesicle residence times. P values are given in Fig. 1f, g, 3f. Scale bar: 2 µm. (b) Single vesicle dot plot for Rab5 vesicles from HeLa cells used in Fig. 1i. P values are given in Fig. 1i. Scale bars: 2 µm. (c) Residence times of vesicles in strains lacking *snx-6* cargo adaptor. Each data point represents a single vesicle that showed kiss-and-run behavior. For a different representation of the data refer to Fig. 6d (n=57 for Rab11_*snx6,* n=62 for Rab5_*snx6*). P values are given in Fig. 6d. (d) Size of Rab5 vesicles in *wild-type* and *snx6(RNAi)* worms (n=6). (e) Size of Rab11 vesicles in *wild-type* and *snx6(RNAi)* worms (n=6). Scale bar: 10 µm.

Supplementary Figure 4

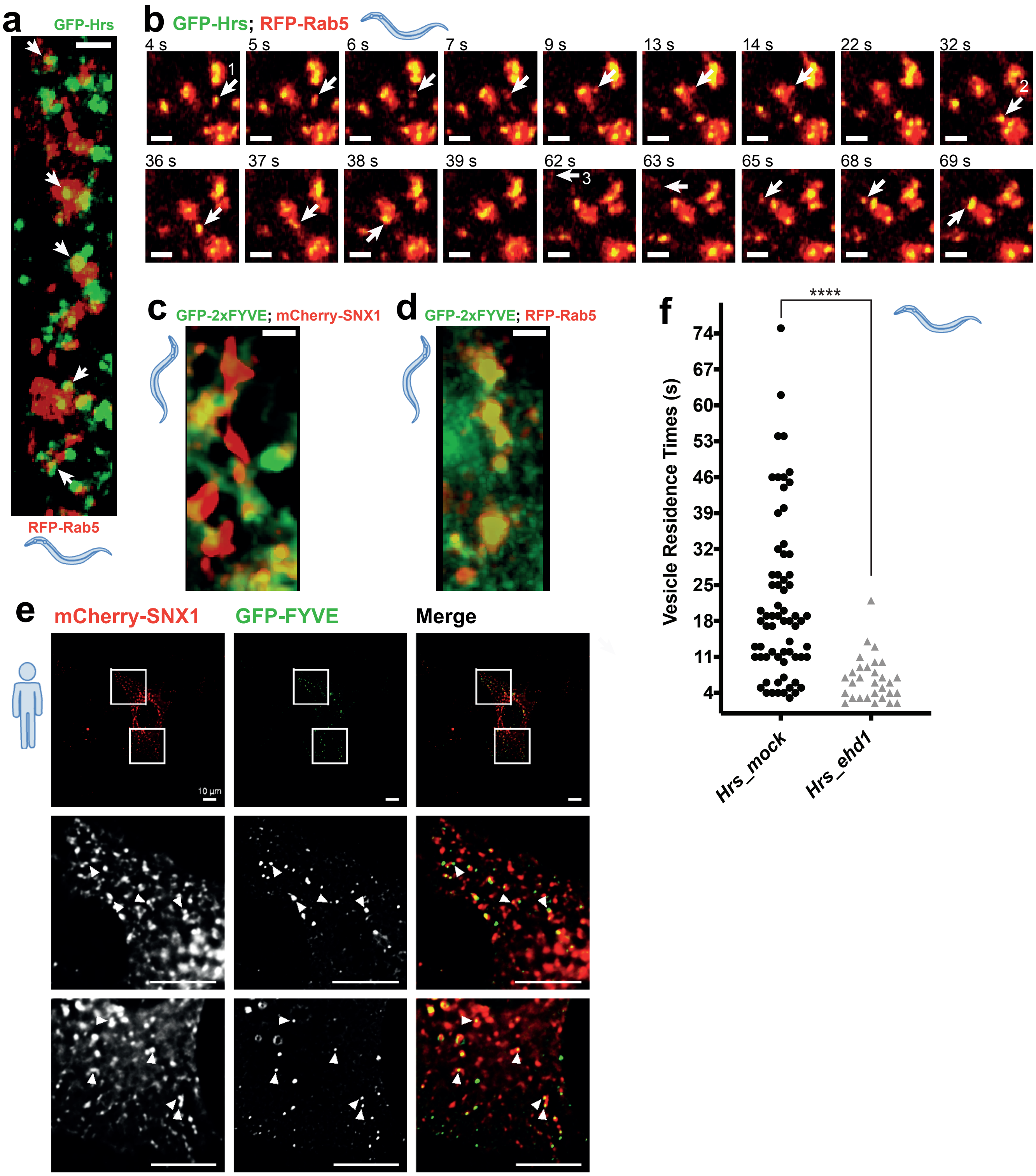

Supplementary Figure 4: (a) ESCRT-0 subunit Hrs can frequently be found on Rab5 compartments (arrows) (3D projection in Supplementary Movie 6) (n=3 experiments with n=20 worms each). (b) Cargo for degradation can potentially be retained on moving Rab5 vesicles by the presence of ESCRT-0 subunit Hrs. Movie stills showing 3 RFP-Rab5 vesicles fusing with a larger sorting endosome (homotypic fusion). Vesicles contain GFP-Hrs domains and move with similar dynamics as small Rab5 vesicles that perform kiss-and-run (see Supplementary Movies 7, 8) (n=12 movies were analyzed from n=66 worms in n=3 experiments). (c) SNX1 compartment in worm intestines only partially co-localize with FYVE marker for PI(3)P. Some (more globular) parts of SNX1 show high PI(3)P content, while other (more tubular) parts seem devoid of PI(3)P (see Supplementary Movie 9) (n=3 experiments with n=17 worms). Scale bars: 2 μm. (d) Rab5 compartments in worm intestines are mostly FYVE positive (i.e., they contain PI(3)P) (see Supplementary Movie 9) (n=3 experiments with n=19 worms). Scale bars: 2 μm. (e) SNX1 compartments in HeLa cells are partially decorated with GFP-FYVE domains, but some SNX1 compartments show no detectable PI(3)P (n=3 experiments with n=20 images each). (f) Single vesicle residence times for Hrs-positive vesicles in worms during kiss-and-run on SNX1 compartments (see also Fig. 2d). P value is given in Fig. 2d.

Supplementary Figure 5

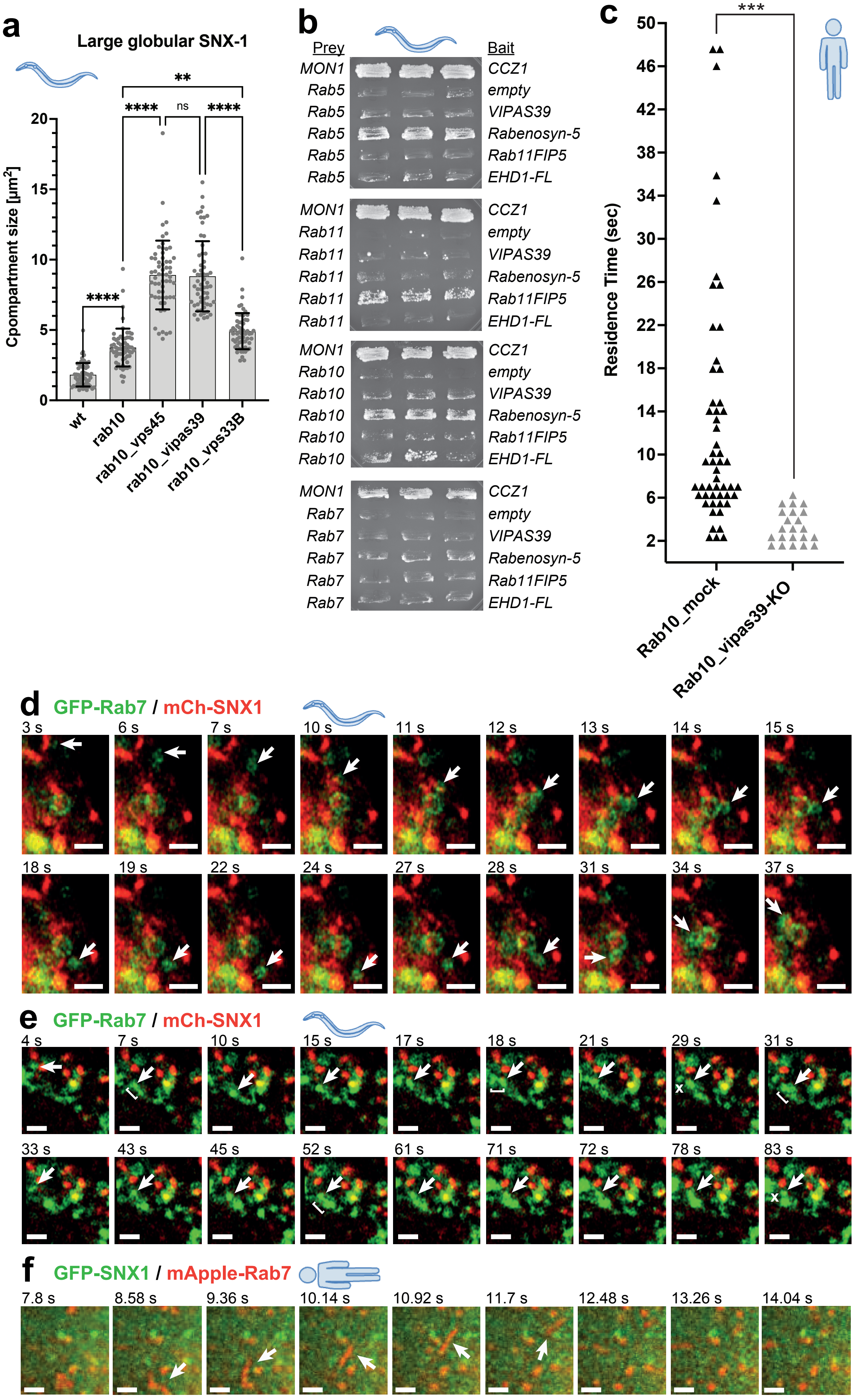

Supplementary Figure 5: (a) Quantification of genetic interaction phenotypes between FERARI subunits and Rab10 (see Fig. 3b). Large globular SNX1 compartments were measured in 6 worms (10 structures each, n=60). Data are presented as mean values +/- SD. One-way ANOVA test was used to determine the statistical significance: wt vs. rab10 P=1.2263E-07, rab10 vs. rab10-vps45 P=5.54E-13, rab10 vs. rab10-vipas39 P=5.54E-13, rab10 vs. rab10-vps33B P=0.004539, rab10-vps45 vs. rab10-vipas39 P=0.998, rab10-vipas39 vs. rab10-vps33B P=5.58E-13. (b) Interactions of *C. elegans* Rab5 and Rab10 with FERARI. Yeast two-hybrid assays showing binding of Rab5 and Rab10 to the RABS-5 subunit of FERARI. Rab11 interacts through Rab11FIP5, while Rab7 shows no interaction. n=6 independent transformants. Quantification of this data is shown in Supplementary Table 2. (c) Single vesicle dot plots of Rab10 residence times in HeLa cells (different representation of graph in Fig. 3g). P value is given in Fig. 3g. (d) Rab7 vesicle (arrow) with no tethering or kiss-and-run with SNX1 compartments (see also Supplementary Movie 13) (n=16 movies with non-sticky interactions from n=3 experiments with n=44 worms). Scale bar: 2 µm. (e) Rab7 homotypic interactions (Supplementary Movie 13). Fusions are indicated by bracket "[" and fissions by "x" (n=13 fusion movies from n=3 experiments with n=44 worms). Scale bar: 2 µm. (f) Rab7 in HeLa cells (arrow) also shows neither tethering nor kiss-and-run behavior with nearby SNX1 compartments (n=3 experiments with n=20 imaged cells each). Scale bar: 2 µm.

Supplementary Figure 6

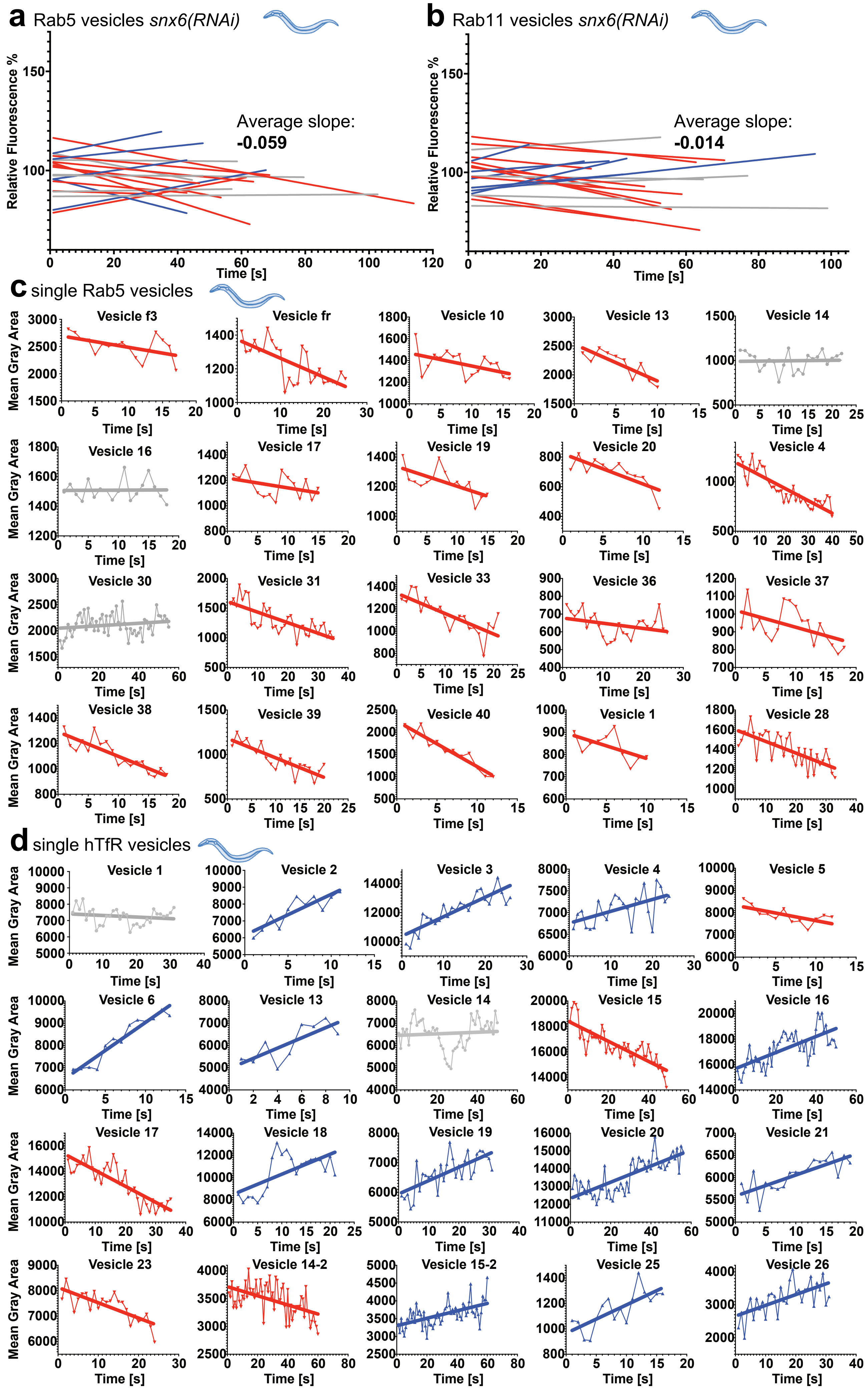

Supplementary Figure 6: (a) Changes in fluorescence intensities in Rab5 vesicles during kiss-and-run. Experiments were carried out in *snx6(RNAi)* worms. Linear regressions are shown for n=20 vesicles. Color coding shows slightly positive slopes in blue and slightly negative slopes in red (flat lines in grey). The average slope was -0.059. (b) Changes in fluorescence intensities in Rab11 vesicles during kiss-and-run. Experiments were carried out in *snx6(RNAi)* worms. Linear regressions are shown for n=20 vesicles. Color coding shows slightly positive slopes in blue and slightly negative slopes in red (flat lines in grey). The average slope was -0.014. (c) Single vesicle fluorescence intensity plots during kiss-and-run docking phase for Rab5 vesicles (see Fig. 5a). (d) Single vesicle fluorescence intensity plots during kiss-and-run docking phase for hTfR-GFP positive vesicles (see Fig. 5c).

Supplementary Figure 7

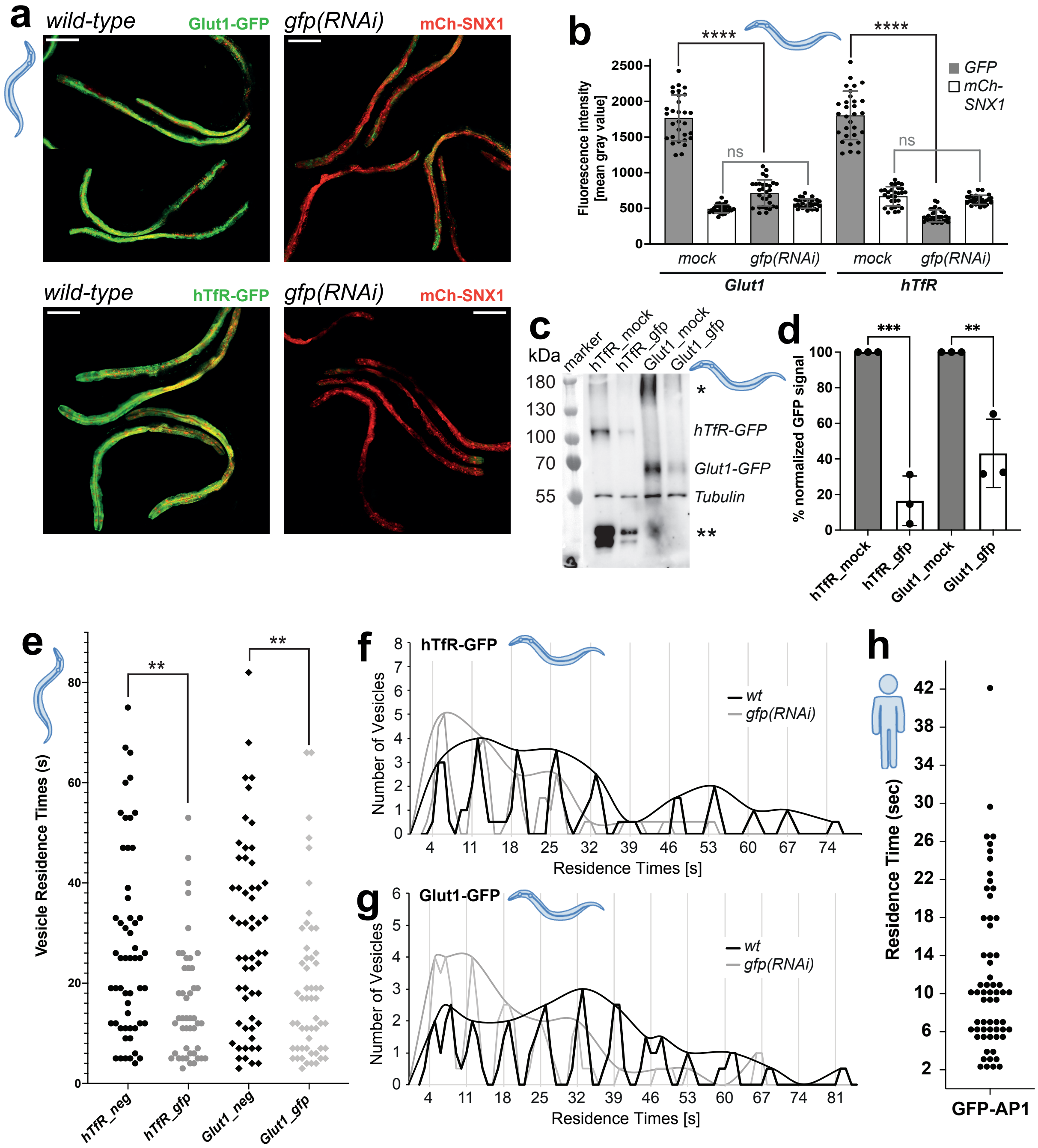

Supplementary Figure 7: (a) Example pictures of worms with mock RNAi and *gfp(RNAi)* that were used for the residence times measurements with overexpressed and reduced cargo in Fig. 5g, h (n=77 hTfR-GFP and n=68 Glut1-GFP worms from n=3 experiments each). Scale bars: 20 µm. (b) Quantification of fluorescence intensity of worms shown in (a), n=30 (3 independent knock-downs). Data are presented as mean values +/- SD. The mCh-SNX1 marker was unaffected by the RNAi and was used as a marker to assess the changes in the GFP markers. One-way multiple comparison ANOVA test P values: Glut1-GFP: *mock vs. gfp(RNAi)* P=2E-15, mCh-SNX1 *mock vs. gfp(RNAi)* P=0.4548, hTfR-GFP: *mock vs. gfp(RNAi)* P=2E-15, mCh-SNX1 *mock vs. gfp(RNAi)* P=0.745. (c) Western blot data showing reduced cargo as in Fig 5g, h. Asterisks indicate either high-molecular-weight bands (*) or lower molecular weight bands (**) that might correspond to post-translational modifications or degradation products, respectively (these bands were not considered in the quantifications) (n=3 independent experiments). (d). Quantification of the western blot data from (d). Data are presented as mean values +/- SD. One-way multiple comparison ANOVA test P values: hTfR-mock vs. hTfR-gfp P=0.0001176, Glut1-mock vs. Glut1-gfp P=0.001681. (e) Dot plots showing residence times for single vesicles doing kiss-and-run. These are the same vesicles used in Fig. 5g, h for the graphs with binning and moving averages, resulting in vesicle groups as peaks. P values are given in Fig. 5g, h. (f) (g), “Bridge” graphs connecting the peaks of Fig. 5g, h to highlight the distribution of vesicles, (f) hTfR-GFP cargo, (g) Glut1-GFP. (h) Single vesicle plot for GFP-AP1 positive vesicles, measuring residence times during kiss-and-run (companion graph to Fig. 8b).

Supplementary Figure 8

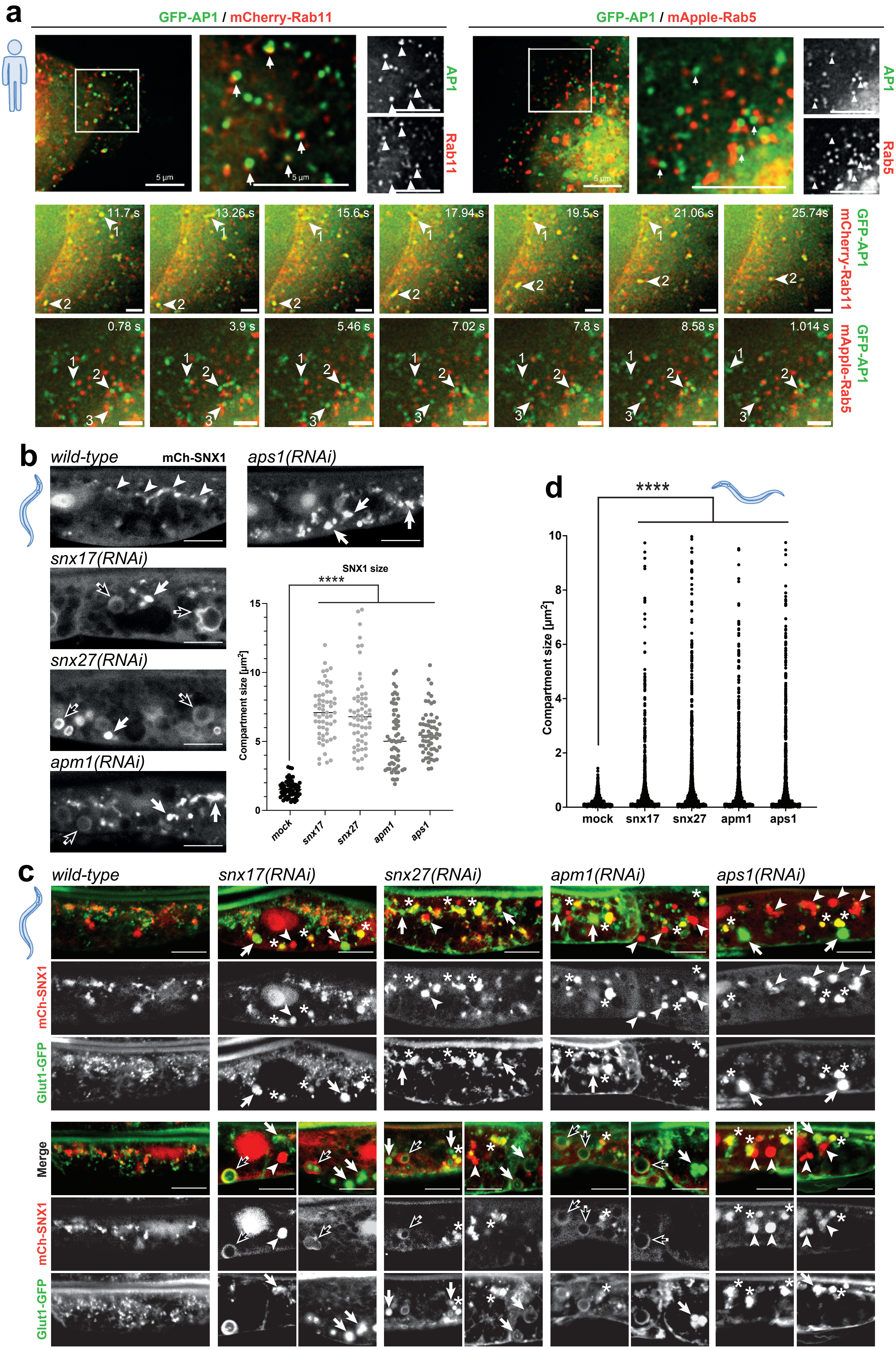

Supplementary Figure 8: (a) HeLa cells transiently co-expressing GFP-AP1 (sigma subunit) together with mCherry-Rab11 or mApple-Rab5. AP1 cargo adaptor co-localizes with Rab11 vesicles but not with Rab5 compartments in HeLa cells (arrows). Stills from Supplementary Movie 20 show 2 moving vesicles labelled with both GFP-AP1 and mCherry-Rab11 (arrowheads 1 and 2 in the upper row). In the lower row, stills show 3 GFP-AP1 vesicles moving independently of mApple-Rab5 (see arrowheads 1-3) (n=3 independent transfection experiments). Scale bars: 5 µm (cells), 2 µm (movie stills). (b) mCherry-SNX1 compartments are enlarged in cargo adaptor knock-down worms. Shown are either large hollow spheres (black arrows) or more irregularly shaped, filled structures (white arrows). The *wild-type* reticulated SNX1 compartments are indicated by arrowheads. Quantification of SNX1 compartment sizes is shown on the right (for n=6 worms and n=10 structures each (n=60)). One-way multiple comparison ANOVA test P values: *mock vs. snx17* P=5.54E-13, *mock vs snx27* P=5.54E-13, *mock vs. apm1* P=6.16E-13, *mock vs. aps1* P=6.03E-13. Scale bar: 10 µm. (c) Adaptor knock-downs affect cargo traffic through SNX1 compartments. RNAi of *snx17, snx27, apm1* or *aps1* cause enlargement of SNX1 compartments (black arrows: large empty round compartments, arrowheads: large filled compartments (see also (D)). The model cargo Glut1-GFP was found in enlarged compartments as well (white arrows). Some of the Glut1 accumulations co-localized with enlarged SNX1 structures (asterisks). Single channel images and merge are shown (n=3 experiments with n=20 worms). Scale bar: 10 µm. (d) Quantification of enlarged Glut1-GFP compartments in adaptor knock-down worms (n=6). One-way multiple comparison ANOVA test P values: *mock vs. snx17* P=2.9853E-08, *mock vs snx27* P=2.9852E-08, *mock vs. apm1* P=2.9852E-08, *mock vs. aps1* P=2.9852E-08.

Supplementary Figure 9

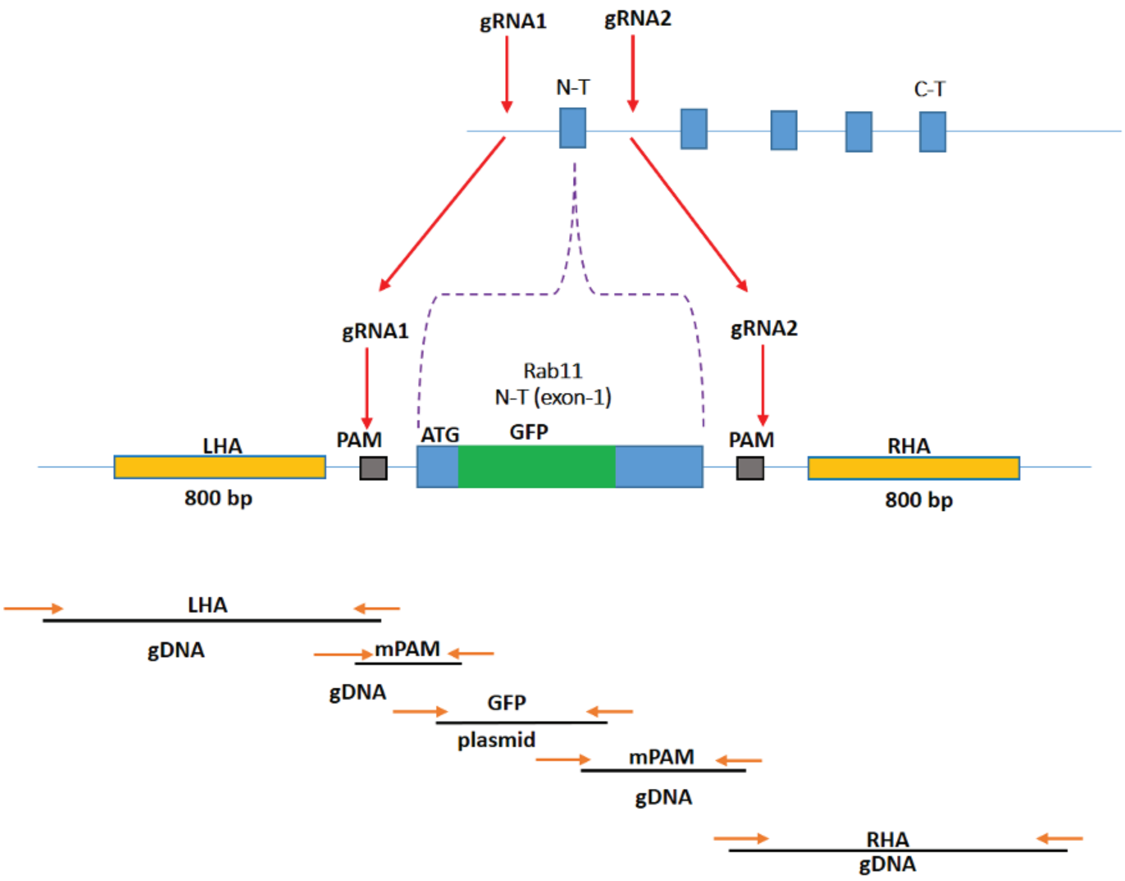

Supplementary Figure 9: Schematic diagram for GFP-Knock in in Rab11 locus.

HLA (Homology left arm); PAM (Protospacer adjacent motif); mPAM (mutated PAM), N-T (N terminus), RHA (Right homology arm); gRNA (guide RNA). Two guide RNAs were designed from introns before and after first exon of Rab11. Annealed oligonucleotides were cloned into two different plasmids containing the mCherry and Puromycin selection marker, respectively. As indicated in the scheme, five PCR products were synthesized. GFP was synthesized by using a GFP containing plasmid as a template and for the rest, genomic DNA from HeLa cells served as template. All these PCR products were cloned into pUC19 plasmid by Gibson assembly. After transformation, sequencing confirmed the insertion of the template into the vector. Two gRNAs containing vectors and the template containing vector were then transfected into HeLa cells. After seven days of transfection, cells were FACS sorted and GFP+ cells were collected. For the confirmation of the GFP-knock-in, western blots were performed.

Supplementary Table 1

guide RNA	
**RAB10**	
guide 1 F	CACCGTGATCGGGGATTCCGGAGTG
guide 1 F R	AAACCACTCCGGAATCCCCGATCAC
guide 2 F	CACCGCATTGCGCCTCTGTAGTAGG
guide 2 R	AAACCCTACTACAGAGGCGCAATGC
**SNX6**	
guide 1 F	CACCGATGATGGTGGGTGTTCTCCG
guide 1 F R	AAACCGGAGAACACCCACCATCATC
guide 2 F	CACCGGTGCAGCGAGGAAACCGAA
guide 2 R	AAACTTCGGTTTCCTCGCTGCACC
**SNX5**	
guide 1 F	CACCGCGACGCGGGACTCGAGCAG
guide 1 R	AAACCTGCTCGAGTCCCGCGTCGC
guide 2 F	CACCGTTTTAAAAAGAACATTCCG
guide 2 R	AAACCGGAATGTTCTTTTTAAAAC

guide RNA for GFP knock-in into Rab11 locus	
guide 1 F	CACCGTTAAGGGGAAGTACTTCCGG
guide 1 R	AAACCCGGAAGTACTTCCCCTTAAC
guide 2 F	CACCGCTCTACACAGTCCTCGTTCG
guide 2 R	AAACCGAACGAGGACTGTGTAGAGC

Primers for GFP knock-in into Rab11 locus	
F	GCATGCCTGCAGGTCGACT TGAAATACTTATGTAAACGGACTTA
R	TTTAAGGGGAAGTACTTCCGGGATCGGCG
F	CGCCGATCCCGGAAGTACTTCCCCTTAAA
R	TCCTCGCCCTTGCTCACCATTGCGCGGCCGAGGAGCGAAA
F	CTCCTCGGCCGCGCAATGGTGAGCAAGGGCGAGGAGCTGTT
R	TACTCGTCGTCGCGGGTGCCCTTGTACAGCTCGTCCATGCCGAGAGTGAT
F	GAGCTGTACAAGGGCACCCGCGACGACGAGTACGACTACC
R	CGGCACGAGGGTCCACCGGGAGTGGCCCGGGTATCCGAAC
F	TGGACCCTCGTGCCGGCCACCCCTGCACTGATATAGGCCT
R	GGTACCCGGGGATCCTCTAGACAAGAAAAGAAAAGGCTAGGTGGGA

Supplementary Table 2: Y2H (yeast two hybrid) interactions

Prey	Bait	Growth
sand-1	*ccz-1*	+++
rab-5	*empty*	-
rab-5	*spe-39*	-
rab-5	*rabs-5*	++
rab-5	*rfip-2*	-
rab-5	*rme-1*	-
sand-1	*ccz-1*	+++
rab-11	*empty*	-
rab-11	*spe-39*	-
rab-11	*rabs-5*	-
rab-11	*rfip-2*	+
rab-11	*rme-1*	-
sand-1	*ccz-1*	+++
rab-10	*empty*	-
rab-10	*spe-39*	(+)
rab-10	*rabs-5*	++
rab-10	*rfip-2*	-
rab-10	*rme-1*	(+)
sand-1	*ccz-1*	+++
rab-7	*empty*	-
rab-7	*spe-39*	-
rab-7	*rabs-5*	-
rab-7	*rfip-2*	-
rab-7	*rme-1*	-

(+) slight growth
