# Supplementary material for: FERARI and cargo adaptors coordinate cargo flow through sorting endosomes
Source: Nat Commun. 2022 Aug 8;13:4620. doi: 10.1038/s41467-022-32377-y (PMC9359993; doi:10.1038/s41467-022-32377-y)
Supplement: Supplementary file 3 — Description of Additional Supplementary Files [file 41467_2022_32377_MOESM3_ESM.pdf]

Description of Additional Supplementary Files

File name: Supplementary Movie 1

Description: Rab11 vesicles in HeLa cells show kiss-and-run behavior. Movies for mock (n=3), vipas39-KO (n=2), ehd1-KO (n=2) and rab11fip5-KO (n=2) are shown. Arrows point to the vesicles with kiss-and-run. Movies are repeated 3 times.

File name: Supplementary Movie 2

Description: Biogenesis of Rab11 vesicles from SNX1 compartments in worms and HeLa cells. Movies for worms (n=3) and HeLa cells (n=2) show tube formation from a larger compartment, followed by pinching-off of a Rab11-positive vesicle (arrows point to the vesicles). Movies are repeated 3 times.

File name: Supplementary Movie 3

Description: Rab5 vesicles show kiss-and-run in worms. Shown are n=2 movies from wild-type (mock) and n=1 movie from ehd1(RNAi) worms. Arrows point to vesicles of interest, in wild-type they show kiss-and-run, in ehd1 worms the vesicle shows little to no tethering. Movies are repeated 3 times.

File name: Supplementary Movie 4

Description: Rab5 vesicles show kiss-and-run in HeLa cells. Shown are n=3 movies for mock and n=3 movies for vipas39-KO cells. Arrows point to vesicles of interest with kiss-and-run. Movies are repeated 3 times.

File name: Supplementary Movie 5

Description: Rab5 vesicles homotypic fusion in worms. Movie with 2 vesicles fusing with each other, then showing kissand-run and finally fusing with a bona fide sorting endosome. Arrows point to the 3 events described. Movie is repeated 3 times.

File name: Supplementary Movie 6

Description: 3D projection of Rab5 vesicles/compartments with colocalizing Hrs signal in worms.

File name: Supplementary Movie 7

Description: Rab5 and Hrs-positive vesicles move together and fuse with larger endosomal structures in worms. Example movie shows n=3 fusion events (arrows) from vesicles into a bona fide sorting endosome. Movie is repeated 3 times.

File name: Supplementary Movie 8

Description: Hrs vesicles show kissand-run in worms. Shown are n=2 mock and n=2 ehd1(RNAi) movies. Arrows point to kiss-andrun events. Movies are repeated 3 times.

File name: Supplementary Movie 9

Description: 3D projections of 2xFYVE marker together with SNX1 and Rab5 in worms.

File name: Supplementary Movie 10

Description: 3D projection of Rab10 vesicles docked to SNX1 tubular structures in worms.

File name: Supplementary Movie 11

Description: Rab10 vesicles show kiss-and-run in worms. Shown are n=3 movies for wildtype(mock) and n=1 movie for ehd1(RNAi) worms. Arrows point to the kiss-and-run events. Movies are repeated 3 times.

File name: Supplementary Movie 12

Description: Rab10 vesicles show kiss-and-run in HeLa cells. Shown are n=3 movies for mock and n=3 movies for vipas39-KO cells. Arrows point to the kissand-run events. Movies are repeated 3 times.

File name: Supplementary Movie 13

Description: Rab7 vesicles do not show kiss-and-run in worms and HeLa cells. Shown is a Rab7 vesicle passing near SNX1 compartments without getting tethered, several Rab7 vesicles combining by fusion into a larger compartment in worms, and moving, but not interacting Rab7 and SNX1 compartments in HeLa cells. Arrows point to the described events. Movies are repeated 3 times.

File name: Supplementary Movie 14

Description: 3D projections of SYX3 networks and their colocalization with SNX1, EHD1 and Rab10 compartments in worms.

File name: Supplementary Movie 15

Description: Cargo transfer from Rab5 vesicles into SNX1 and from SNX1 into Rab11 vesicles in HeLa cells. Co-localizing cargo is shown in white. Arrows point to kiss-and-run events, n=2 movies are shown for Rab5 and Rab11, respectively. Movies are repeated 3 times.

File name: Supplementary Movie 16

Description: 3D projections of Rab5 vesicles docking onto SNX1 tubular structures in mock and snx6(RNAi) worms.

File name: Supplementary Movie 17

Description: 3D projections of Rab11 vesicles docking onto SNX1 tubular structures in mock and snx6(RNAi) worms.

File name: Supplementary Movie 18

Description: 3D projections of Rab10 vesicles docking onto SNX1 tubular structures or EHD1 compartments in mock and snx6(RNAi) worms.

File name: Supplementary Movie 19

Description: AP1 vesicles show kiss-and-run in HeLa cells. Movie shows 2 vesicles with kiss-andrun events (indicated by arrows). Movie is repeated 3 times.

File name: Supplementary Movie 20

Description: AP1 and Rab11 move together on vesicles in HeLa cells. Movies from n=2 cells show n=6 vesicles that are labeled with AP1 and Rab11 and move together. Movies with Rab5 vesicles show no co-localization or concerted movement of AP1 and Rab5. Movies are repeated 3 times.
