# Supplementary material for: FERARI and cargo adaptors coordinate cargo flow through sorting endosomes
Source: Nat Commun. 2022 Aug 8;13:4620. doi: 10.1038/s41467-022-32377-y (PMC9359993; doi:10.1038/s41467-022-32377-y)
Supplement: Supplementary file 24 — Reporting Summary [file 41467_2022_32377_MOESM24_ESM.pdf]

nature portfolio

Corresponding author(s): Anne Spang

Last updated by author(s): 26/07/2022

Reporting Summary

Nature Portfolio wishes to improve the reproducibility of the work that we publish. This form provides structure for consistency and transparency in reporting. For further information on Nature Portfolio policies, see our Editorial Policies and the Editorial Policy Checklist.

Statistics

For all statistical analyses, confirm that the following items are present in the figure legend, table legend, main text, or Methods section.

n/a	Confirmed	
☐	☒	The exact sample size (*n*) for each experimental group/condition, given as a discrete number and unit of measurement
☐	☒	A statement on whether measurements were taken from distinct samples or whether the same sample was measured repeatedly
☐	☒	The statistical test(s) used AND whether they are one- or two-sided<br>*Only common tests should be described solely by name; describe more complex techniques in the Methods section.*
☒	☐	A description of all covariates tested
☒	☐	A description of any assumptions or corrections, such as tests of normality and adjustment for multiple comparisons
☐	☒	A full description of the statistical parameters including central tendency (e.g. means) or other basic estimates (e.g. regression coefficient) AND variation (e.g. standard deviation) or associated estimates of uncertainty (e.g. confidence intervals)
☐	☒	For null hypothesis testing, the test statistic (e.g. *F*, *t*, *r*) with confidence intervals, effect sizes, degrees of freedom and *P* value noted<br>*Give P values as exact values whenever suitable.*
☒	☐	For Bayesian analysis, information on the choice of priors and Markov chain Monte Carlo settings
☒	☐	For hierarchical and complex designs, identification of the appropriate level for tests and full reporting of outcomes
☐	☒	Estimates of effect sizes (e.g. Cohen's *d*, Pearson's *r*), indicating how they were calculated

Our web collection on statistics for biologists contains articles on many of the points above.

Software and code

Policy information about availability of computer code

Data collection	Software microscopy - ZEN Black Version 2.3 SP1 FP3 (LSM 880 Airyscan), ZEN 2.3 (Blue edition; widefield microscope), FV3000 (FV31S-SW Version 2.5.1) system software (Olympus Fluoview 3000)<br>Western Blots: on film or alternatively captured on a FUSION FX Vilber Lourmat using FusionCapt Advance
Data analysis	Fiji and plugins therein.<br>Statistical analysis data from Western Blots and microscopy - GraphPad Prism 7 and 8 and Excel

For manuscripts utilizing custom algorithms or software that are central to the research but not yet described in published literature, software must be made available to editors and reviewers. We strongly encourage code deposition in a community repository (e.g. GitHub). See the Nature Portfolio guidelines for submitting code & software for further information.

Data

Policy information about availability of data

All manuscripts must include a data availability statement. This statement should provide the following information, where applicable:

- Accession codes, unique identifiers, or web links for publicly available datasets
- A description of any restrictions on data availability
- For clinical datasets or third party data, please ensure that the statement adheres to our policy

The authors declare that the main data supporting the findings of this study are available within the article and its Supplementary Information files. The script used for analysis is available at https://doi.org/10.5281/zenodo.6907356

Field-specific reporting

Please select the one below that is the best fit for your research. If you are not sure, read the appropriate sections before making your selection.

☒ Life sciences ☐ Behavioural & social sciences ☐ Ecological, evolutionary & environmental sciences

For a reference copy of the document with all sections, see nature.com/documents/nr-reporting-summary-flat.pdf

Life sciences study design

All studies must disclose on these points even when the disclosure is negative.

Sample size	Sample size was chosen in each case according to experimental design. All cell biology experiments were performed at least in 3 independent biological repeats. For C. elegans experiments, >19 worms were analyzed stemming from at least 3 independent replicates. Exact numbers are given in the figure legends.
Data exclusions	No data was excluded from any analyses reported in this study.
Replication	At least 3 independent experiments were performed per condition. All experiments were shown to be reproducible.
Randomization	No randomization was used. All the samples were prepared with known composition and contained appropriate controls, which mitigates the influence of co-variates.
Blinding	Blinding was not used in this study as phenotypes were obvious.

Reporting for specific materials, systems and methods

We require information from authors about some types of materials, experimental systems and methods used in many studies. Here, indicate whether each material, system or method listed is relevant to your study. If you are not sure if a list item applies to your research, read the appropriate section before selecting a response.

Materials & experimental systems

n/a	Involved in the study	
☐	☒	Antibodies
☐	☒	Eukaryotic cell lines
☒	☐	Palaeontology and archaeology
☐	☒	Animals and other organisms
☒	☐	Human research participants
☒	☐	Clinical data
☒	☐	Dual use research of concern

Methods

n/a	Involved in the study	
☒	☐	ChIP-seq
☒	☐	Flow cytometry
☒	☐	MRI-based neuroimaging

Antibodies

Antibodies used	All antibodies are described in the manuscript. The following antibodies were used in this study: polyclonal. Polyclonal rabbit anti-GFP (TP401; Torrey Pines; 1:2,000 for western blotting and 1:200 for immunostaining), Rab10 (D36C4) Rabbit mAb (#8127, Cell signaling, 1:1000 for western blotting), Rab11 Anti-Rab11 antibody (ab3612, abcam, 1:1000 for western blotting), Recombinant Anti-SNX5 antibody (ab180520, abcam, 1:1000 for western blotting). Purified Mouse Anti-SNX1 (611482, BD biosciences, 1:100 for IF), SNX6 mouse monoclonal antibody (D-5) (sc-365965, Santa Cruz, 1:1000 for western blotting), 1:500 anti-Giantin (rabbit) antibody (BioLegend cat 924302). For pulldowns, Trap beads (nanobodies) were used. GFP-Trap_A (chromotek, gta-20) was used for GFP pulldowns. HRP-conjugated goat anti-mouse IgG (H+L) secondary antibody (Thermo Fisher Scientific; 31430; 1:10,000) and polyclonal HRP-conjugated goat-anti-rabbit IgG (Thermo Fisher Scientific; 31460; 1:10,000) were used (incubated for 1h at room temperature) to detect bound antibodies with Blotting detection kit WesternBrightTM ECL (advansta; K-12045-D50). Alexa Fluor 488–goat anti-rabbit IgG (H+L) (Invitrogen; A-11034) and Alexa Fluor 594–goat anti-mouse IgG (H+L) cross-adsorbed secondary antibodies (Invitrogen; R37121) were used for immunofluorescence.
Validation	All primary antibodies are commercially available and validated by the manufacturer. In addition, most of the antibodies have been used by other research groups previously as referenced in the associated manuscript. The antibodies against VIPAS39, VPS45, Rab11FIP5 and rabenosyn5 have been independently verified in the Western blot analysis in wild-type and CRISPR-Cas9 gene-edited cell lines, in which the corresponding gene was deleted.<br><br>anti-GFP TP401 from Torrey Pines was validated by<br>Lionnard L, Duc P, Brennan M, Kueh A, Pal M, Guardia F, et al. TRIM17 and TRIM28 antagonistically regulate the ubiquitination and anti-apoptotic activity of BCL2A1. Cell Death Differ. 2019;26:902-917<br>Liu C, Lin C, Gao C, May Simera H, Swaroop A, Li T. Null and hypomorph Prickle1 alleles in mice phenocopy human Robinow syndrome and disrupt signaling downstream of Wnt5a. Biol Open. 2014;3:861-70

And 74 further papers cited on https://www.labome.com/product/Torrey-Pines-Biolabs/TP401.html

Rab10 (D36C4) Rabbit mAb (#8127, Cell signaling) was validated by the company for western blots in various cell lines and for IF inMCF7 cells (see https://www.cellsignal.com/products/primary-antibodies/rab10-d36c4-xp-rabbit-mab/8127)
Used in publications:
Tu H, Zhang ZW, Qiu L, Lin Y, Jiang M, Chia SY, Wei Y, Ng ASL, Reynolds R, Tan EK, Zeng L. Increased expression of pathological markers in Parkinson's disease dementia post-mortem brains compared to dementia with Lewy bodies. BMC Neurosci. 2022 Jan 4;23(1):3. doi: 10.1186/s12868-021-00687-4. PMID: 34983390; PMCID: PMC8725407.
Tu H, Zhang ZW, Qiu L, Lin Y, Jiang M, Chia SY, Wei Y, Ng ASL, Reynolds R, Tan EK, Zeng L. Increased expression of pathological markers in Parkinson's disease dementia post-mortem brains compared to dementia with Lewy bodies. BMC Neurosci. 2022 Jan 4;23(1):3. doi: 10.1186/s12868-021-00687-4. PMID: 34983390; PMCID: PMC8725407.
and 38 additional publication found on the company web page linked above.

Rab11 Anti-Rab11 antibody (ab3612, abcam) was validated by the company on
https://www.abcam.com/rab11b-antibody-ab3612.html#lb
for western blots in MDCK cells and PC3 cell extract and for IF in HeLa cells.
Used in Publications:
Wang H et al. Unraveling GLUT-mediated transcytosis pathway of glycosylated nanodisks. Asian J Pharm Sci 16:120-128 (2021).
Howe EN et al. Rab11b-mediated integrin recycling promotes brain metastatic adaptation and outgrowth. Nat Commun 11:3017 (2020).
and 27 more cited on the website (see link above).

Recombinant Anti-SNX5 antibody (ab180520, abcam)
https://www.abcam.com/snx5-antibody-epr14358-ab180520.html
was validated by the company for western blots in mouse tissues and jurkat cells, for IF in rat and mouse kidney preparations and 293T cells.
Used in publications:
Evans AJ et al. Acute inactivation of retromer and ESCPE-1 leads to time-resolved defects in endosomal cargo sorting. J Cell Sci 133:N/A (2020).
Han J et al. Involvement of CASP9 (caspase 9) in IGF2R/CI-MPR endosomal transport. Autophagy N/A:1-17 (2020).
and 3 more found on the company website above.

Purified Mouse Anti-SNX1 (611482, BD biosciences)
was validated by the company for western blots and IF in HeLa cells:
https://www.bdbiosciences.com/zh-cn/products/reagents/microscopy-imaging-reagents/immunofluorescence-reagents/purified-mouse-anti-snx1.611482
Used in publications:
Cozier GE, Carlton J, McGregor AH. The phox homology (PX) domain-dependent, 3-phosphoinositide-mediated association of sorting nexin-1 with an early sorting endosomal compartment is required for its ability to regulate epidermal growth factor receptor degradation. J Biol Chem. 2002; 277(50):48730-48736. (Clone-specific: Immunofluorescence, Western blot).
Haft CR, de la Luz Sierra M, Barr VA, Haft DH, Taylor SI. Identification of a family of sorting nexin molecules and characterization of their association with receptors. Mol Cell Biol. 1998;18(12):7278-7287. (Biology).
and 2 additional publications cited on the company website (see link above).

SNX6 mouse monoclonal antibody (D-5) (sc-365965, Santa Cruz)
was validated by the company for western blots in several cell lines (including HeLa) and for IF in human tissue samples
https://www.scbt.com/p/snx6-antibody-d-5
Used in publications:
Olson-Wood MG, Jorgenson LM, Ouellette SP, Rucks EA. Inclusion Membrane Growth and Composition Are Altered by Overexpression of Specific Inclusion Membrane Proteins in Chlamydia trachomatis L2. Infect Immun. 2021 Jun 16;89(7):e0009421. doi: 10.1128/IAI.00094-21. Epub 2021 Jun 16. PMID: 33875478; PMCID: PMC8208519.
Evans AJ, Daly JL, Anuar ANK, Simonetti B, Cullen PJ. Acute inactivation of retromer and ESCPE-1 leads to time-resolved defects in endosomal cargo sorting. J Cell Sci. 2020 Aug 3;133(15):jcs246033. doi: 10.1242/jcs.246033. PMID: 32747499; PMCID: PMC7420817.
and 5 additional papers cited on the website (link above).

HRP-conjugated goat anti-mouse IgG (H+L) secondary antibody (Thermo Fisher Scientific ; 31430)
was validated by the manufacturer
https://www.thermofisher.com/antibody/product/Goat-anti-Mouse-IgG-H-L-Secondary-Antibody-Polyclonal/31430
for western blots and immunoprecipitations in various cell lines and tissue lysates.
Was used in publications:
SARS-CoV-2 pseudovirus enters the host cells through spike protein-CD147 in an Arf6-dependent manner. In Emerging Microbes Infections on 1 December 2022 by Zhou, Y. Q., Wang, K., et al.. Applications: WB
KLF5-induced miR-487a augments the progression of osteosarcoma cells by targeting NKX3-1 in vitro. In Oncology Letters on 1 August 2022 by Luo, A., Liu, H., et al.. Applications: WB
and 1367 additional citations found on the company website (see link above).

HRP-conjugated goat-anti-rabbit IgG (Thermo Fisher Scientific; 31460)
https://www.thermofisher.com/antibody/product/Goat-anti-Rabbit-IgG-H-L-Secondary-Antibody-Polyclonal/31460
was validated by the manufacturer for western blots, IF and immunoprecipitations assays in various human and mouse cell lines (including HeLa).
Used in publications:
SARS-CoV-2 pseudovirus enters the host cells through spike protein-CD147 in an Arf6-dependent manner. In Emerging Microbes Infections on 1 December 2022 by Zhou, Y. Q., Wang, K., et al.. Applications: WB
In vitro analyses of paracrine effects of murine classically activated macrophage on beige adipocyte metabolism. In STAR Protocols on 16 September 2022 by Yao, J., Wu, D., et al..
and 1712 additional publications cited on the manufacturer's website (see link above).

Alexa Fluor 488–goat anti-rabbit IgG (H+L) (Invitrogen; A-11034)
was validated by the company in 43 different IF experiments (including HeLa cells).
https://www.thermofisher.com/antibody/product/Goat-anti-Rabbit-IgG-H-L-Highly-Cross-Adsorbed-Secondary-Antibody-Polyclonal/A-11034
Used in 4777 publications (see website above for details):
Plasmodium SAS4: basal body component of male cell which is dispensable for parasite transmission. In Life Science Alliance on 1 September 2022 by Zeeshan, M., Brady, D., et al..
Ferrostatin1 alleviates oxalateinduced renal tubular epithelial cell injury, fibrosis and calcium oxalate stone formation by inhibiting ferroptosis. In Molecular Medicine Reports on 1 August 2022 by Xie, J., Ye, Z., et al..

Alexa Fluor 594–goat anti-mouse IgG (H+L) cross-adsorbed secondary antibodies (Invitrogen; R37121)
was validated by the manufacturer for HeLa cells IF
https://www.thermofisher.com/antibody/product/Goat-anti-Mouse-IgG-H-L-Cross-Adsorbed-Secondary-Antibody-Polyclonal/R37121
and used in 97 publications cited on the company website (see link above):
Diversity of bacterial small RNAs drives competitive strategies for a mutual chaperone. In Nature Communications on 4 May 2022 by Roca, J., Santiago-Frangos, A., et al..
The role of long noncoding RNA Nron in atherosclerosis development and plaque stability. In IScience on 18 March 2022 by Du, M., Wang, C., et al..

anti-Giantin (rabbit) antibody (BioLegend cat 924302)
https://www.biolegend.com/fr-ch/products/purified-anti-giantin-antibody-11365
was tested for activity in IF by the manufacturer
Used in 20 publications:
https://www.citeab.com/antibodies/2864094-924302-anti-giantin-antibody/publications
NPC1 regulates the distribution of phosphatidylinositol 4-kinases at Golgi and lysosomal membranes. In The EMBO Journal on 1 July 2021 by Kutchukian, C., Vivas, O., et al..
A weak COPI binding motif in the cytoplasmic tail of SARS-CoV-2 spike glycoprotein is necessary for its cleavage, glycosylation, and localization. In FEBS Letters on 1 July 2021 by Jennings, B. C., Kornfeld, S., et al..

Eukaryotic cell lines

Policy information about cell lines

Cell line source(s)	HeLa CCL2 and HEK 293 kind gift Prof Martin Spiess.
Authentication	Standard cell lines, authenticated by ATCC. Recently (2021), the cell lines' identities authenticated by STR analysis by Microsynth AG (Balgach Switzerland).
Mycoplasma contamination	We routinely test our cell lines for mycoplasma contamination. All cell lines were negative.
Commonly misidentified lines (See ICLAC register)	No commonly misidentified cell lines were used

Animals and other organisms

Policy information about studies involving animals; ARRIVE guidelines recommended for reporting animal research

Laboratory animals	C. elegans nematodes were grown to the young adult stage, N2 wild-type strain background was used, the following worm strains and transgenes were used in this study: pwIs206[vha6p::GFP::rab-10 + Cb unc-119(+)], pwIs782[Pvha-6::mCherry::SNX-1], pwIs414[Pvha-6::RFP::rab-10, Cb unc-119(+)], dkIs218[Popt-2-GFP-syx-3; Cb unc-119(+)], pwIs621[vha-6::mCherry-RME-1], pwIs72[vha6p::GFP::rab-5 + unc-119(+)], pwIs170[vha6p::GFP::rab-7 + Cb unc-119(+)], pwIs90[Pvha-6::hTfR-GFP; Cbr-unc-119(+)], qxEx2247 [Pvha-6::Glut1::GFP], pwIs69[vha6p::GFP::rab-11 + unc-119(+)], pwIs87[Pvha-6::GFP::rme-1; Cbr-unc-119(+)], [Pdhs-3::dhs-3::GFP], pwIs481[Pvha-6::mans-GFP, Cbr-unc-119(+)], pwIs518[vha-6::GFP-HGRS-1], pwIs846[Pvha-6-RFP-rab-5; Cb unc-119(+)], rab-10(ok1494). All experiments were carried out with hermaphrodites.
Wild animals	No wild animals were used in the study.
Field-collected samples	No field collected samples were used in this study.
Ethics oversight	Non-vertebrate model system. No ethical oversight required.

Note that full information on the approval of the study protocol must also be provided in the manuscript.
