# Supplementary material for: FERARI and cargo adaptors coordinate cargo flow through sorting endosomes
Source: Nat Commun. 2022 Aug 8;13:4620. doi: 10.1038/s41467-022-32377-y (PMC9359993; doi:10.1038/s41467-022-32377-y)
Supplement: Supplementary file 25 — Source Data [file 41467_2022_32377_MOESM25_ESM.zip › Source Data/source data Western blot.pdf]

Fig. 3C

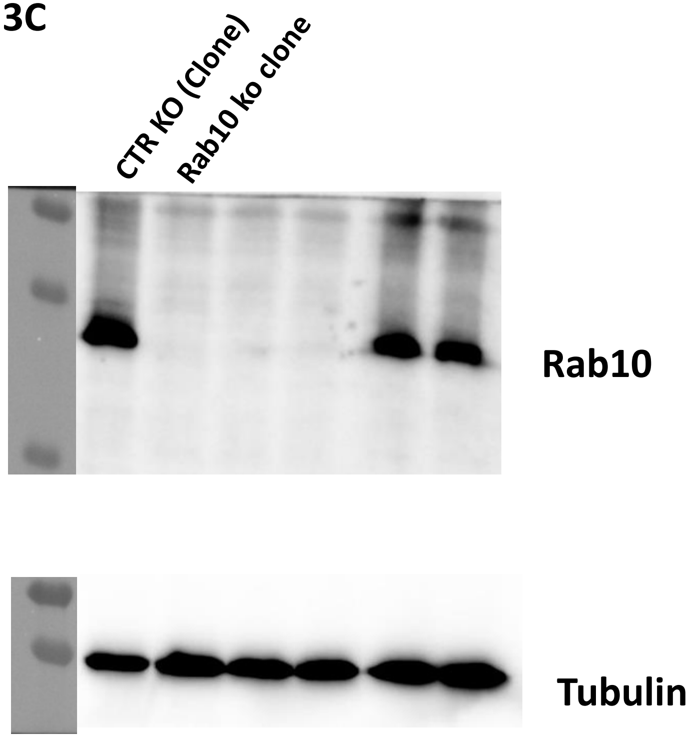

Fig. 7f (left)

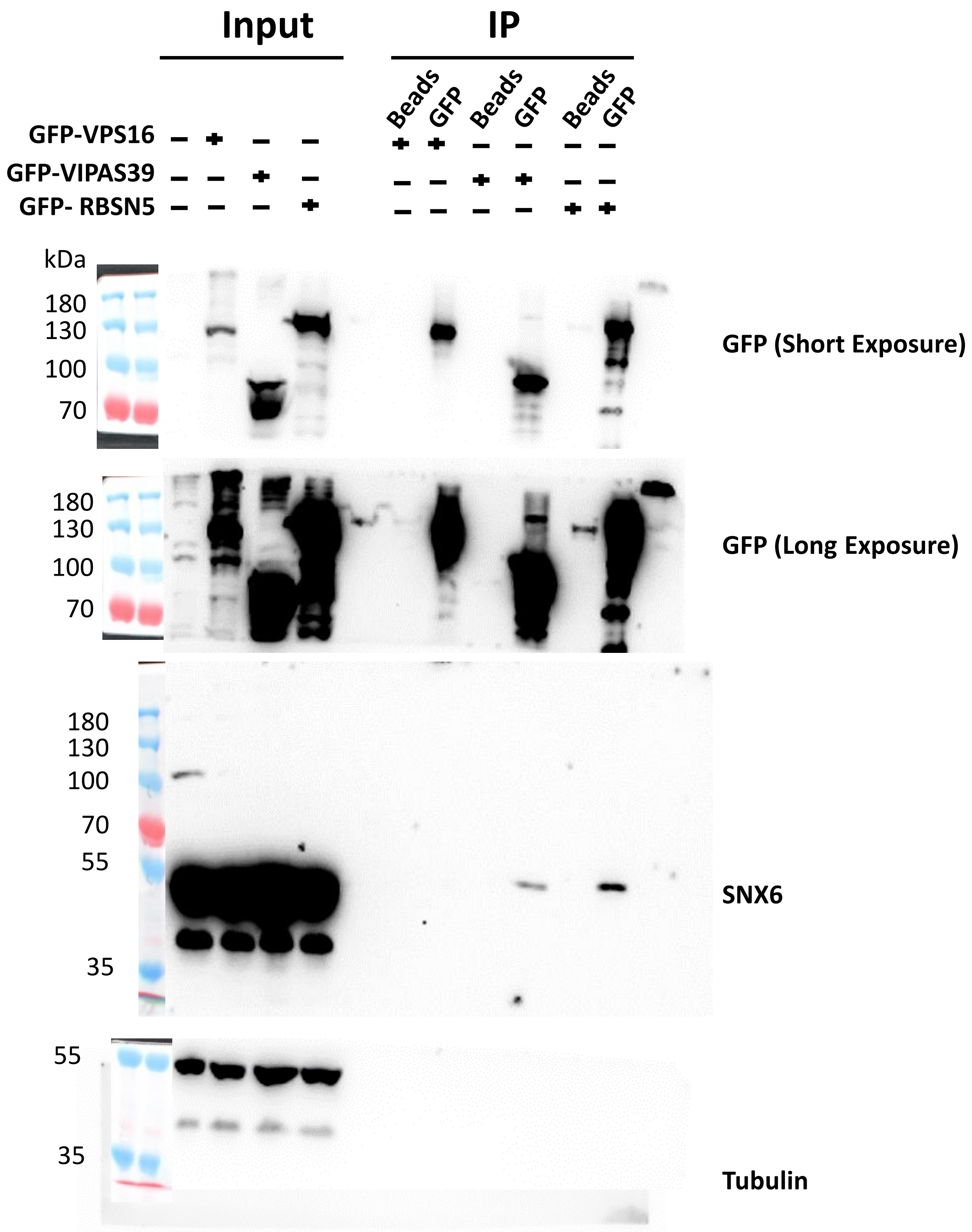

Input
IP
Beads
GFP
Beads
GFP
Beads
GFP
GFP-VPS16
GFP-VIPAS39
GFP- RBSN5
kDa
180
130
100
70
55
35
GFP (Short Exposure)
GFP (Long Exposure)
SNX6
Tubulin

Fig. 7F (right)

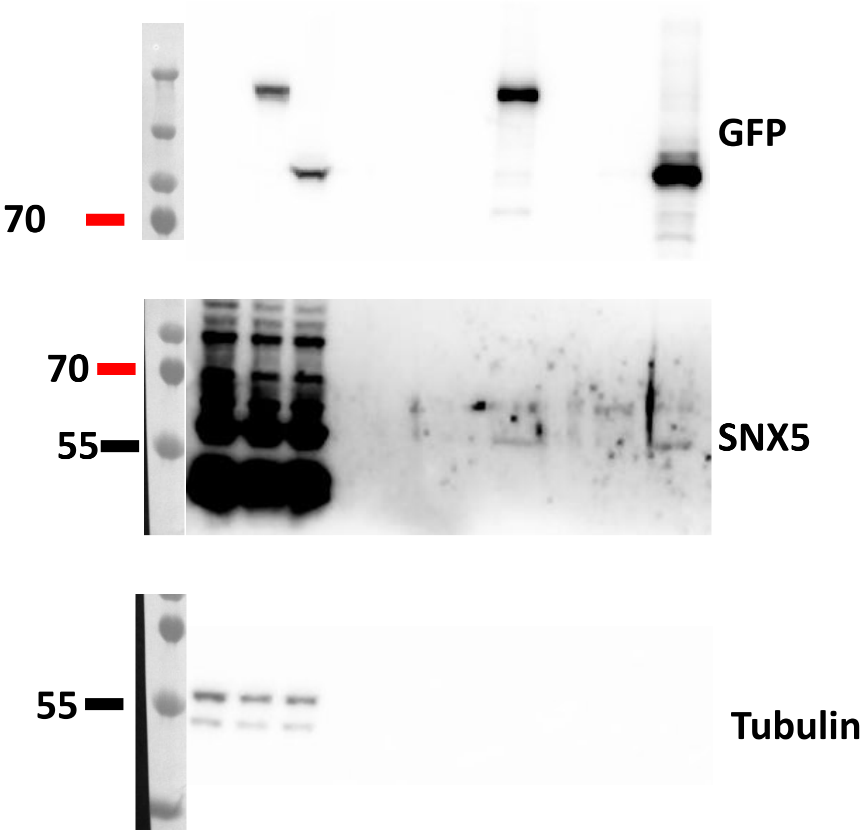

Supplementary Fig. 1B (left panel)

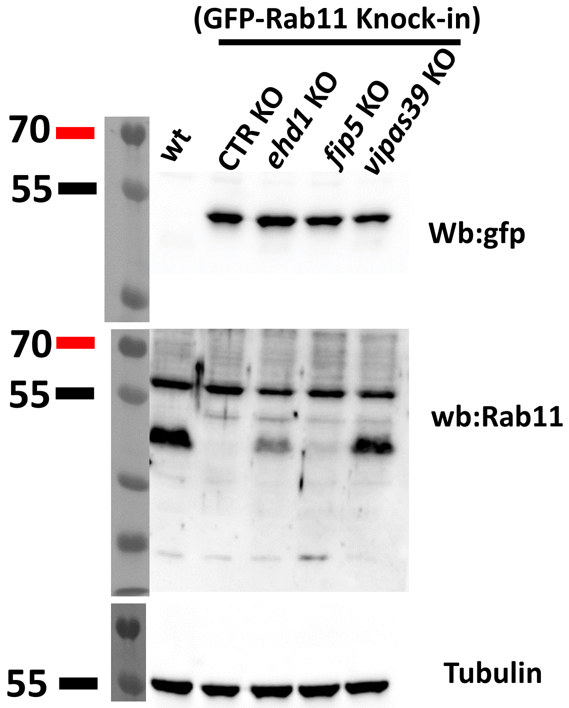

Supplementary Fig. 1B (middle panel)

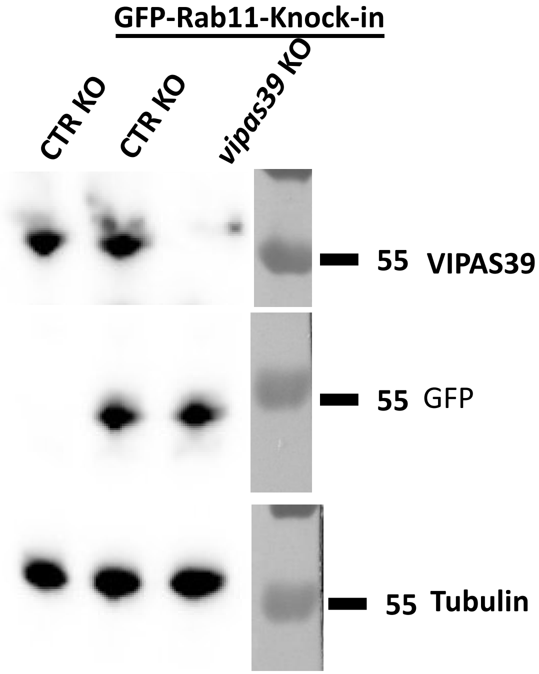

Supplementary Fig. 1B (right panel)

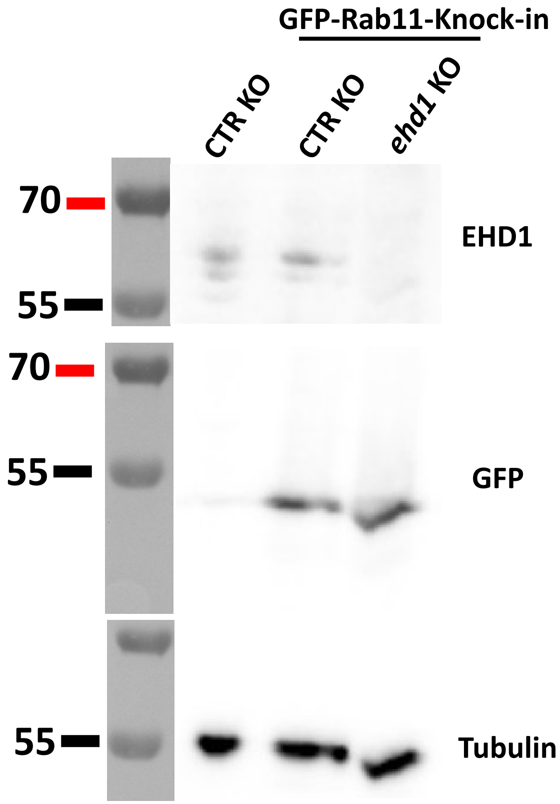

Supplementary Fig. 1B (middle lower panel)

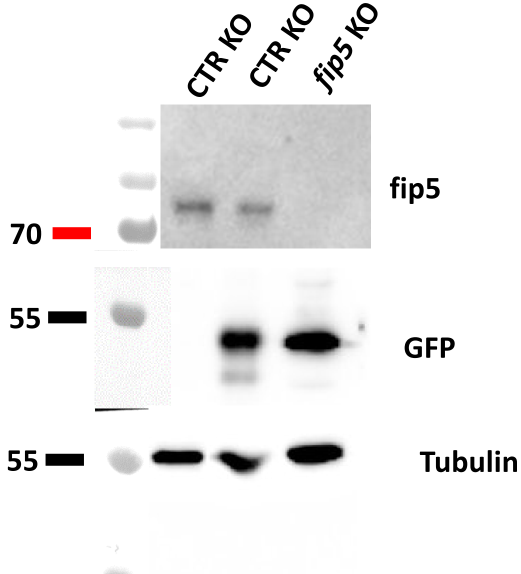

Supplementary Fig. 1C

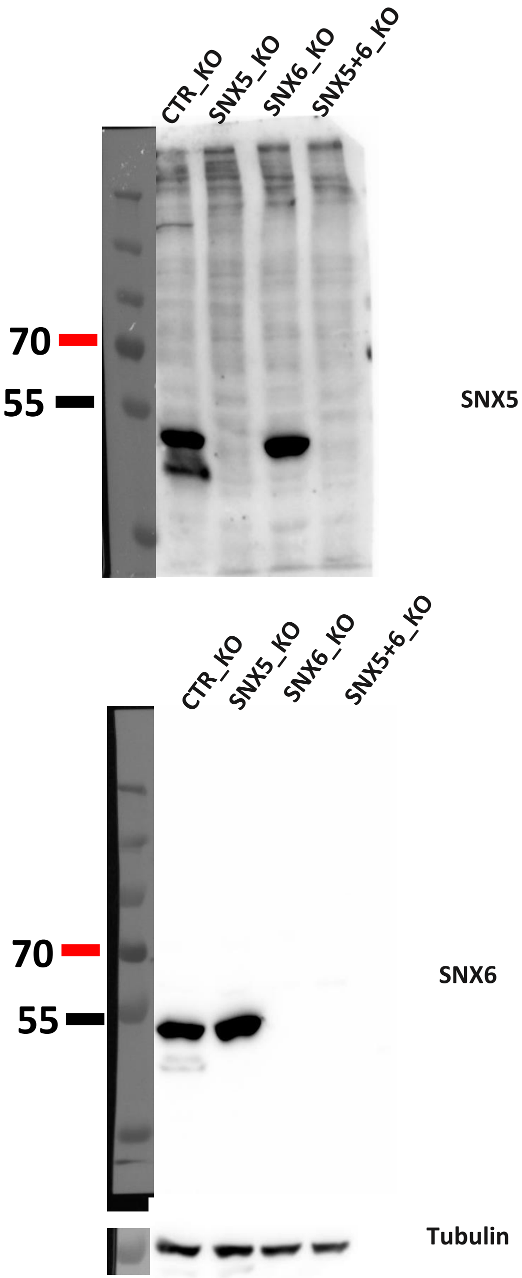

Supplementary Fig. 1E

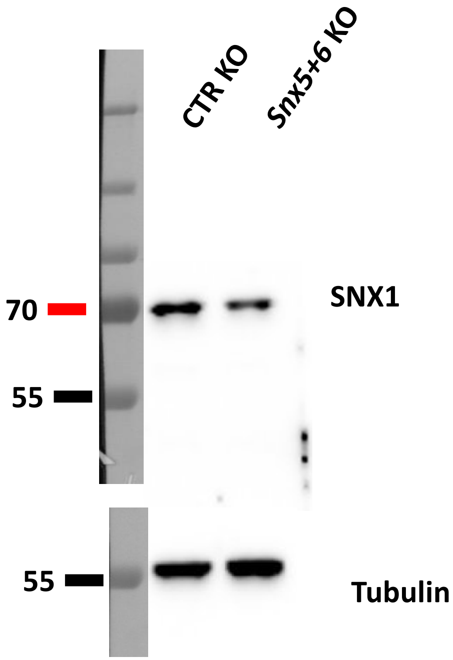

Fig. S7c

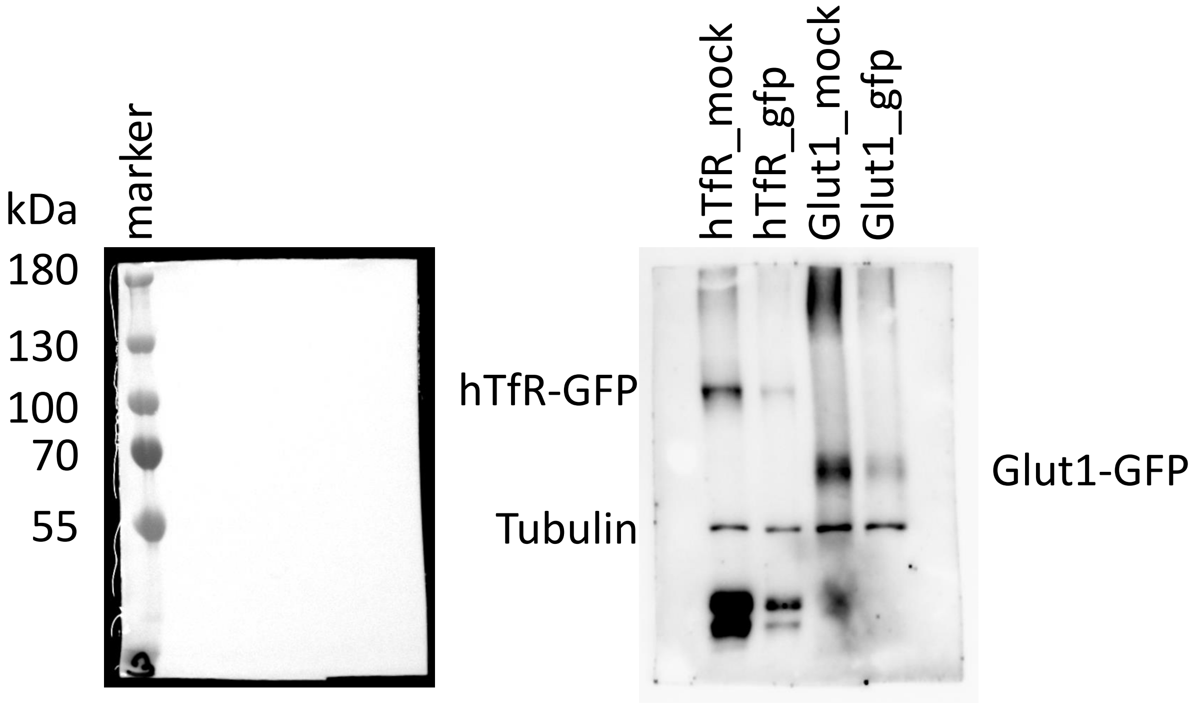
marker
kDa
180
130
100
70
55
hTfR_mock
hTfR_gfp
Glut1_mock
Glut1_gfp
hTfR-GFP
Tubulin
Glut1-GFP
